# Zebrafish *myo7aa* affects congenital hearing by regulating Rho-GTPase signaling

**DOI:** 10.3389/fnmol.2024.1405109

**Published:** 2024-07-15

**Authors:** Binling Xie, Jiaxin Liang, Jifan Jiang, Ting Zeng, Ling Liu, Dinghua Xie, Ganghua Zhu, Lei Xiong, Kanjia Zhang, Dong Liu, Jie Gong, Xiangding Chen, Ruosha Lai, Huaping Xie

**Affiliations:** ^1^Laboratory of Animal Nutrition and Human Health, Hunan International Joint Laboratory of Animal Intestinal Ecology and Health, College of Life Science, Hunan Normal University, Changsha, Hunan, China; ^2^State Key Laboratory of Developmental Biology of Freshwater Fish, College of Life Science, Hunan Normal University, Changsha, Hunan, China; ^3^Laboratory of Molecular and Statistical Genetics, College of Life Sciences, Hunan Normal University, Changsha, China; ^4^Department of Otorhinolaryngology—Head & Neck Surgery, The Second Xiangya Hospital of Central South University, Changsha, Hunan, China; ^5^Nantong Laboratory of Development and Diseases, Key Laboratory of Neuroregeneration of Jiangsu and MOE, School of Life Sciences, Co-innovation Center of Neuroregeneration, Nantong University, Nantong, Jiangsu, China

**Keywords:** *myo7aa*, Rho GTPase signaling, hearing, zebrafish, CRISPR/Cas9

## Abstract

**Introduction:**

*myo7aa*, the homolog of the human Usher 1B syndrome pathogenic gene, *myo7A*, plays an important role in stereociliary development and maintenance, therefore, is critical for hearing and balance. However, the molecular mechanisms that *myo7aa* regulate hearing and balance still need to be studied.

**Methods:**

In this study, we generated two independent zebrafish *myo7aa* knockout lines using CRISPR/Cas9 technology. To investigate the effects of *myo7aa* on hearing, YO-PRO-1 staining and startle response assay were used. To gain insight into the specific molecular mechanisms by which *myo7aa* affects hearing, transcriptome sequencing and bioinformatics analysis were employed.

**Results:**

Our study showed that hair cells of *myo7aa*-/- zebrafish can not take up YO-PRO-1 fluorescent dye and are insensitive to acoustic stimulation in *myo7aa*-/- zebrafish compared to wild type. Genes related to the Rho GTPase signaling pathway, such as arhgap33, dab2ip, and arghef40, are significantly down-regulated in *myo7aa*-/- zebrafish embryos at 3 dpf. GTP and ATP compensation can partially rescue the hair cell defects in *myo7aa* knockout zebrafish.

**Discussion:**

Our findings suggest that zebrafish *myo7aa* affects congenital hearing by regulating Rho GTPase signaling, and loss of *myo7aa* leads to abnormal Rho GTPase signaling and impairs hair cell function. *myo7aa*, *myo7A*, arhgap33, dab2ip, arghef40 and *myo7aa*-/- fonts in the abstract are italicized. -/- is a superscript format.

## Introduction

Deafness and hearing impairment are major global health problems. The ear is an important hearing and balance organ for humans, and the inner ear hair cells are the key sensory cells for auditory conversion.

There are about 15,000 hair cells in the human inner ear, among which there are about 3,000 cochlear hair cells as auditory receptors (Rao et al., 2019). When the hair cell is stimulated, the apical stereociliary deflection occurs, leading to the opening of the mechanical conduction channel at the tip of the stereociliary, K^+^ influx, and membrane potential changes. The calcium channel at the hair cell synapse opens, and the synaptic vesicle fusing and releasing neurotransmitters to transmit sound signals to the brain (Fettiplace, 2017; Nicolson, 2017). Zebrafish is widely used in the auditory research because of its embryonic transparency, morphological structure and physiological function of inner ear and lateral line hair cells, which are similar to those of human cochlear hair cells (Pickett and Raible, 2019).

More than 160 different MYO7A mutations have been found to cause a variety of human hearing disorders, including Usher 1B syndrome, recessive nonsyndromic deafness (DFNB2), and dominant nonsyndromic deafness (DFNA11) (Ma et al., 2016).

The MYO7A protein in zebrafish is highly homologous to the proteins found in human and mice. All the interaction domains and signaling protein domains of the MYO7A protein are evolutionary conserved. The *myo7aa* gene in zebrafish is located on chromosome 18 and spans a length of 96,803 bp, with 49 exons. It encodes one MYSc protein domain, four IQ motifs, two MyTH4_B41 tandem domains, and one SH3 protein domain. Mariner zebrafish is the first fish model associated with human hereditary deafness (Ernest et al., 2000). Five alleles of Mariner have been identified by forward genetic screening methods. Mariner zebrafish larvae are characterized by defects in inner ear hair cell bundle, reduced sensitivity to acoustic vibration, and reduced or absent extracellular hair cell potential. These characteristics are similar to the phenotype of shaker-1 mice (Self et al., 1998).

Stereocilia are actin-based cell processes on the apical surface of hair cells in the inner ear, which play a key role in hearing and balance sensation. Stereocilia development and maintenance are tightly regulated, and defects in this process often lead to hearing or balance disorders. The Rho GTPase family consists of about 20 members, including CDC42, RAC1, RhoA, and others, which play important roles in cytoskeleton rearrangement, cell motility, cell polarity, axon guidance, vesicle trafficking, and cell cycle progression (Heasman and Ridley, 2008). RhoA regulates F-actin formation and adhesion through its downstream effectors, such as mDia (Watanabe et al., 1999). The Rho GTPase cell division cycle 42 (CDC42) is located in hair cell stereocilia and is responsible for filopodia formation and actin stress fiber assembly (Du et al., 2021; Dai et al., 2023). *Rac1* regulates the interaction between kinocilium and stereocilia via p21-activated kinase (PAK), inducing membrane folding and lamellipodia formation, which are required for cohesion of developing hair bundles. Loss of *Rac1* in mouse ear epithelium leads to abnormal cochlear epithelial morphology and reduced number of auditory hair cells. Hair cells also exhibit defects in planar cell polarity and morphogenesis of stereociliary bundles, including bundle fragmentation or deformation (Grimsley-Myers et al., 2009).

In this study, we utilized CRISPR/Cas9 technology to generate zebrafish *myo7aa* gene knockout lines and examined the role of *myo7aa* in sensory hair cells. The lack of YO-PRO-1 fluorescence staining and the startle response assay indicated the knockout of *myo7aa* affected the normal function of the hair cells. Comparative transcriptome analysis showed significant changes in the expression levels of genes involved in endocytosis and exocytosis and Rho GTPase signaling in *myo7aa* mutant larvae. GTP and ATP compensation can partially rescue the hair cell defect in *myo7aa* knockout zebrafish. Taken together, our findings suggest that loss of *myo7aa* leads to aberrant Rho GTPase signaling, which is essential for normal hair cell function.

## Materials and methods

### Zebrafish maintenance and husbandry

Tübingen (TU) zebrafish (Laboratory of Animal Nutrition and Human Health, College of Life Science, Hunan Normal University) was used in this study. The embryos were were obtained through natural mating and incubated at 28.5°C. Additionally, the embryos were cultured in E3 water (286.7 mg NaCl, 12.7 mg KCl, 48.3 mg CaCl_2_·2H_2_O, 81.7 mg MgSO4·7H_2_O, 50 μL 0.01% methylene blue per liter of pure water) in Petri dishes until day 5. When developed to desired stages, embryos were collected and fixed with 4% paraformaldehyde (PFA) in phosphate buffered saline (PBS) overnight at 4°C.

### CRISPR-Cas9 knockout of *myo7aa*

The *myo7aa* mutant lines were generated with the CRISPR/Cas9 system. Firstly, we obtained the mRNA sequence and amino acid sequence of *myo7aa* gene from NCBI database and analyzed the structural domain of MYO7AA protein on SMART website.^1^ Then, We searched the intron and exon sequences of zebrafish *myo7aa* using the UCSC (UCSC Genome Browser Home) database, and designed two target sites in exon 6, which is located in the coding region of the MYSc structural domain: 5’-GTATACGGGGTCCATCTTAGTGGG-3′ and 5’-GAATAGCAGTTGTCTGCGACGGG-3′. The T7 promoter (TAATACGACTCACTATA) was added at the 5′ end of the target sequence, and the upstream sequence of the sgRNA backbone sequence (GTTTTTAGAGCTAGAAATAG) was added at the 3′ end of the target sequence as the forward priming sequence, and the downstream sequence of the sgRNA backbone as the reverse priming sequence for PCR amplification. The sgRNA was synthesized *in vitro* using the T7 Transcription Kit (Thermo Fisher Scientific). sgRNA1 and sgRNA2 were purified and recovered using the RNA Purification Kit (Qiagen). Cas9 protein was purchased from Thermo Fisher Scientific. Finally, the two sgRNAs and Cas9 protein were prepared and mixed together (final concentration was 20 μg/μL sgRNA and 500 μg/μL Cas9 protein). Approximately 1 nL of the mixture was injected into wild-type embryos at the single-cell stage of zebrafish embryos. After injection, embryos were incubated at 28.5°C.

### Genotyping

A pair of primers for genotyping was designed at both ends of the two target sites: *myo7aa-Fwd-GT* (TGTTGCAATGTCCTGATGGG) and *myo7aa-Rev-GT* (ACAGCATATCGTCCCATGGA). For wild-type, the PCR product is 361 bp. The targeted genomic regions were amplified by PCR and subjected to 1.5% agarose gel electrophoresis to determine the success of the knockdown.

Control and injected embryos were randomly selected for validity testing at 36 hpf. The two target sites are 142 bp apart, if both target sites work well, there will be a deletion of approximately 142 bp compared to the control. Once the mutation was confirmed in the injected embryos, the remaining fish were cultured to 45 dpf, then each fish was tail clipped, genomic DNA was extracted for genotyping, and the fish with the ~142 bp deletion were cultured to adulthood as the F0 generation and then crossed to WT. Two independently inherited heterozygous mutant zebrafish lines, the PCR product sizes of line1 and line2 are 247 and 475 bp, respectively, were obtained from the offspring of the same F0 generation mutant. Two PCR products with sizes of 247 bp and 475 bp were purified and sent to Qingke Biotech for Sanger sequencing.

### *In situ* hybridization

The mRNA sequence of the *myo7aa* gene was obtained from NCBI, and the primer sequence for anti-sense RNA probe was designed using the primer3 input online primer design software. The primers were then sent to Sangon Biological Company for synthesis. The primers used for the *in situ* hybridization probes in this study were as follows: *myo7aa* Fwd (GTATACGGGGTCCATCTTAGT) and *myo7aa* Rvs (CATCAGATCCTTCAAGTCCAC). The reverse primer added to the T7 core promoter sequence. Zebrafish embryos at the desired stage were fixed in 4% paraformaldehyde (PFA) overnight and then analyzed by WISH as described previously (Chitramuthu and Bennett, 2013). A digoxigenin UTP-labeled antisense RNA probe for *myo7aa* was generated by an *in vitro* transcription method using T7 RNA polymerase (Thermo Fisher, Waltham, MA, United States).

### Hair cell staining of zebrafish

Twenty control and mutant embryos that developed to 60 hpf were stained overnight with YO-PRO-1 dye at a dilution of 1:2000 in E3 water. The next day, the embryos were washed with E3 water three times at 5-min intervals. The zebrafish were then anesthetized with 0.4% MS-222 (tricaine) solution, and 1% low-melting-point agarose was added to the laser confocal petri dish. All fluorescence imaging was conducted using a Zeiss LSM 900 laser scanning confocal microscope. Z-stacks were analyzed using ImageJ and brightness and contrast were equally adjusted for all relevant control and experimental images.

### Quantitative real time polymerase chain reaction

Homozygous mutant embryos were screened by YO-PRO-1 staining, and the total RNA was extracted from control and mutant embryos at 3 dpf using trizol reagent. The cDNA was synthesized using a reverse transcription kit (TaKaRa) with random primers. Real-time quantitative PCR reactions (qPCR) were performed on a QuantStudio 3 real-time PCR system (Thermo Fisher Scientific) using a SYBR kit (Vazyme). *β-actin* was used as the reference gene, and the data were analyzed using the (2^-△△Ct^) method (t-test, *p* < 0.05).

### Startle response of zebrafish larvae

Five days after fertilization (dpf) of 20 larvae, the experiment was conducted in a petri dish with a layer of thin embryo culture medium. To provide sound stimuli, a mini-vibrator was attached to the bottom of the Petri dish. The acoustic stimuli used a frequency of 600 Hz and an intensity of 9 dB re.1 ms^−2^, and were applied for duration of 30 ms (Wei et al., 2022; Wang et al., 2023). For each stimulus level, this was repeated 20 times with a 3-min interval. The behavioral responses of the larvae to sound stimuli were also recorded by an infrared camera over a period of 6 s. Then, the movement trajectory of the larvae was extracted from the recorded film. The average movement distance and peak velocity were analyzed as typical parameters to quantify the startle response of larvae to sound stimuli.

In this experiment, we tested wild-type zebrafish larvae, *myo7aa*^−/−^zebrafish larvae, ATP-treated zebrafish larvae, and GTP-treated zebrafish larvae. The concentration of ATP was 3 mM, and the concentration of GTP treatment was 4 mM. The treatments were initiated 2 h prior to the test and remained in either ATP or GTP solution throughout the duration of the test.

### RNA-seq analysis

Total RNA was extracted using the mirVana miRNA Isolation Kit (Ambion) and the Agilent 2,100 Bioanalyzer (Agilent Technologies, Santa Clara, CA, United States) was used to evaluate RNA integrity. The samples with RNA Integrity Number (RIN) ≥ 7 were subjected to the subsequent analysis. According to the manufacturer’s instructions, the TruSeq Stranded mRNA LTSample Prep Kit (Illumina, San Diego, CA, USA) was used to construct libraries. These libraries were then sequenced on the Illumina sequencing platform (HiSeqTM 2,500 or Illumina HiSeq X Ten) to generate 125 bp/150 bp paired-end reads. After the raw data had been processed by Trimmomatic, the cleaned reads were mapped into the GRCz11 reference genome using Hisat2. The FPKM value of each transcript was calculated using Ballgown, and the FPKM of each transcript is calculated using Cufflinks. The differentially expressed transcripts were filtered with *p*-value <0.05 and | log_2_ fold change | > 1 as thresholds.

### Bioinformatics analysis

Differential expression analysis between *myo7aa* knockout group and control group was performed using R (v3.6.1) and RStudio (v4.3.0), package DESeq2 (v1.24.0). Genes with Padj (FDR) < 0.05 were considered as significantly differentially expressed genes. The results of differentially expressed genes were heatmapped using the pheatmapR package. Gene ontology (GO) analysis is a common method for large-scale functional enrichment analysis. GO database can annotate genes, and gene products can be enriched from three aspects: molecular function (MF), biological process (BP) and cellular component (CC) Gene Ontology Consortium (2015). GO biological process analysis was performed using clusterProfiler package (Yu et al., 2012). Item screening criteria was adjusted *p*-value <0.05 was considered statistically significant, and the *p*-value correction method was Benjamini Hochberg (BH). The results were visualized by Sangerbox, a bioinformatics analysis website, and the important key genes in the pathways were shown. KEGG and reactome pathway enrichment was performed by KOBAS (v3.0) (Kanehisa and Goto, 2000; Xie et al., 2011; Kanehisa, 2019; Kanehisa et al., 2021), transcript annotations were retrieved using the Bioconductor org.Dr.eg.db package, and ggplot2 (Wickham, 2016) package was used to visualize the correlation analysis results.

## Results

### Spatiotemporal expression pattern of *myo7aa*

**Figure 1:**
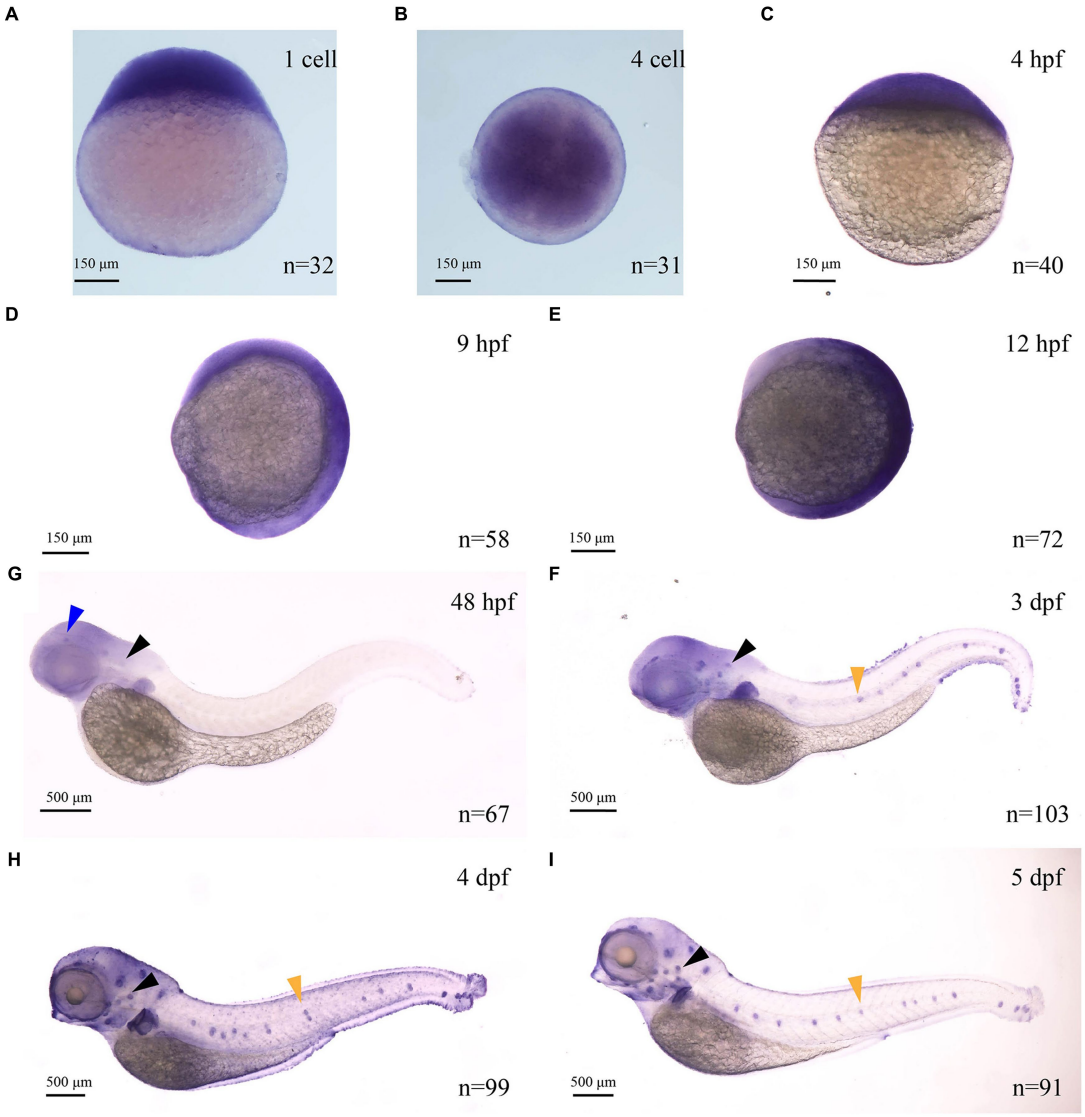
The expression of *myo7aa* at different stages **(A–I)**, arrowheads indicate zebrafish retina (blue), inner ear (black) and lateral line hair cells (yellow); n: the number of embryos.

Zebrafish *myo7aa* was the ortholog of the human deafness pathogenic gene *MYO7A*. In mice and zebrafish, MYO7A is present in sensory hair cells, regions rich in F-actin (Wasfy et al., 2014). It has been reported in the literature that *myo7aa* is expressed in the maculae of the ellipsoid capsule and the globular capsule in zebrafish embryos developing to 24 hpf and 32 hpf (Schwarzer et al., 2017). However, the spatiotemporal expression pattern of this gene in zebrafish is still not well understood. To investigate the spatiotemporal expression model of this gene, we synthesized a digoxigenin-labeled antisense RNA probe of *myo7aa*. *In situ* hybridization results showed that the *myo7aa* is maternally expressed at the 1-cell stage (Figure 1A), and at the 4-cell stage (Figure 1B). It is ubiquitously expressed at 4 hpf (Figure 1C), 9 hpf (Figure 1D), and 12 hpf (Figure 1E). *myo7aa* was specifically expressed in the hair cells of the inner ear and retina at 48 hpf (Figure 1F), in the hair cells of inner ear, retina and lateral line at 3 dpf (Figure 1G), and strongly expressed in the hair cells of inner ear, retina and lateral line at 4 dpf and 5 dpf (Figures 1H,I). These results further suggest that zebrafish *myo7aa* may affect hair cell development and loss of *myo7aa* may lead to hearing impairment.

### Establishment of *myo7aa* knockout lines

**Figure 2:**
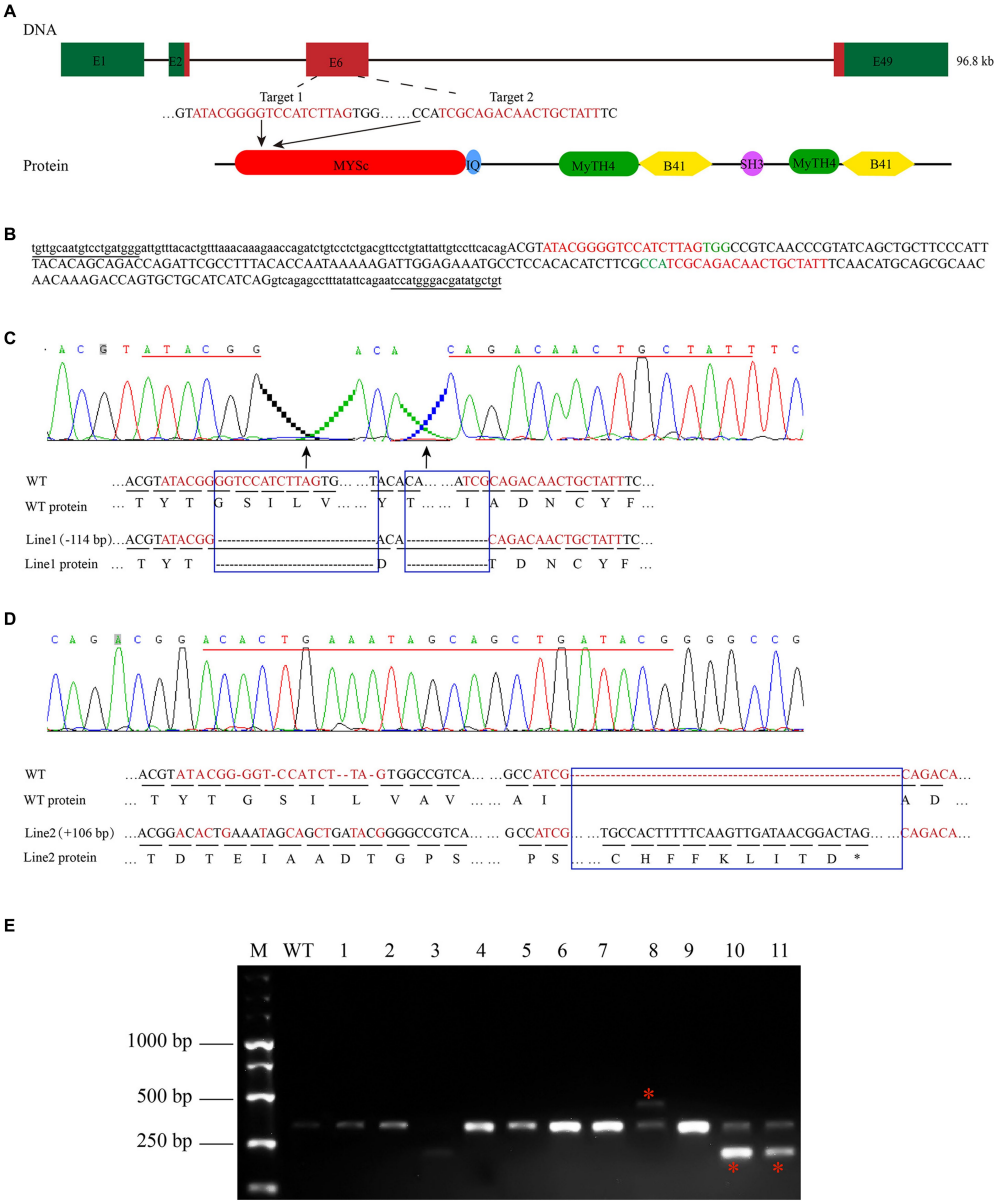
Zebrafish *myo7aa* gene knockout. **(A)** Schematic representation of the CRISPR/Cas9 target region in *myo7aa* gene. Green box represents the 3′UTR and 5′UTR, while the red box indicates exons. **(B)** Sequence of the CRSIPR/Cas9 target region in the *myo7aa* gene. Underlined: detection primers; red font: target site sequence; green font: PAM sequence; capital letters: exon sequence; lowercase letters: intron sequences. **(C,D)** Sequencing peak map, genomic DNA sequence, and amino acid sequence between two independent mutant alleles. Compared with the wild type, the *myo7aa* line1 mutation had a 114 bp deletion and the line2 mutation had a 106 bp insertion. The sequence of the target site is shown in red. Rectangular boxes indicate deletion or insertion sites in the mutant. ^*^Indicates termination of protein translation. **(E)** The screening results of the F1 generation adult fish showed the presence of DNA marker, wild type fish, and mutant fish.

To study the effect of the zebrafish *myo7aa* gene on hair cells, zebrafish *myo7aa* knockout lines that can be stably inherited by CRISPR/Cas9 technology were generated. The target site was designed in exon 6 of the MYSc domain coding region (Figure 2A), and genotyping primers for detecting wild-type PCR products were designed at both ends with a length of 361 bp (Figure 2B). Two independently inherited heterozygous mutant zebrafish lines, line1 and line2, with 114 bp deletion (Figure 2C) and 104 bp insertion (Figure 2D), were selected from the offspring of the same F0 generation mutant (Figure 2E). Line1 mutant caused a 38-amino acid deletion in the MYSc domain of MYO7AA protein (Figure 2C), and line2 mutant caused a frameshift mutation in the open reading frame of *myo7aa*, leading to premature termination of protein translation (Figure 2D). The *myo7aa* homozygous mutant embryos showed no obvious defects in body size during early development (not shown), but the homozygous juveniles of both mutant lines died at 10–12 days.

### The *myo7aa* mutation leads to abnormal hair cell function in zebrafish

**Figure 3:**
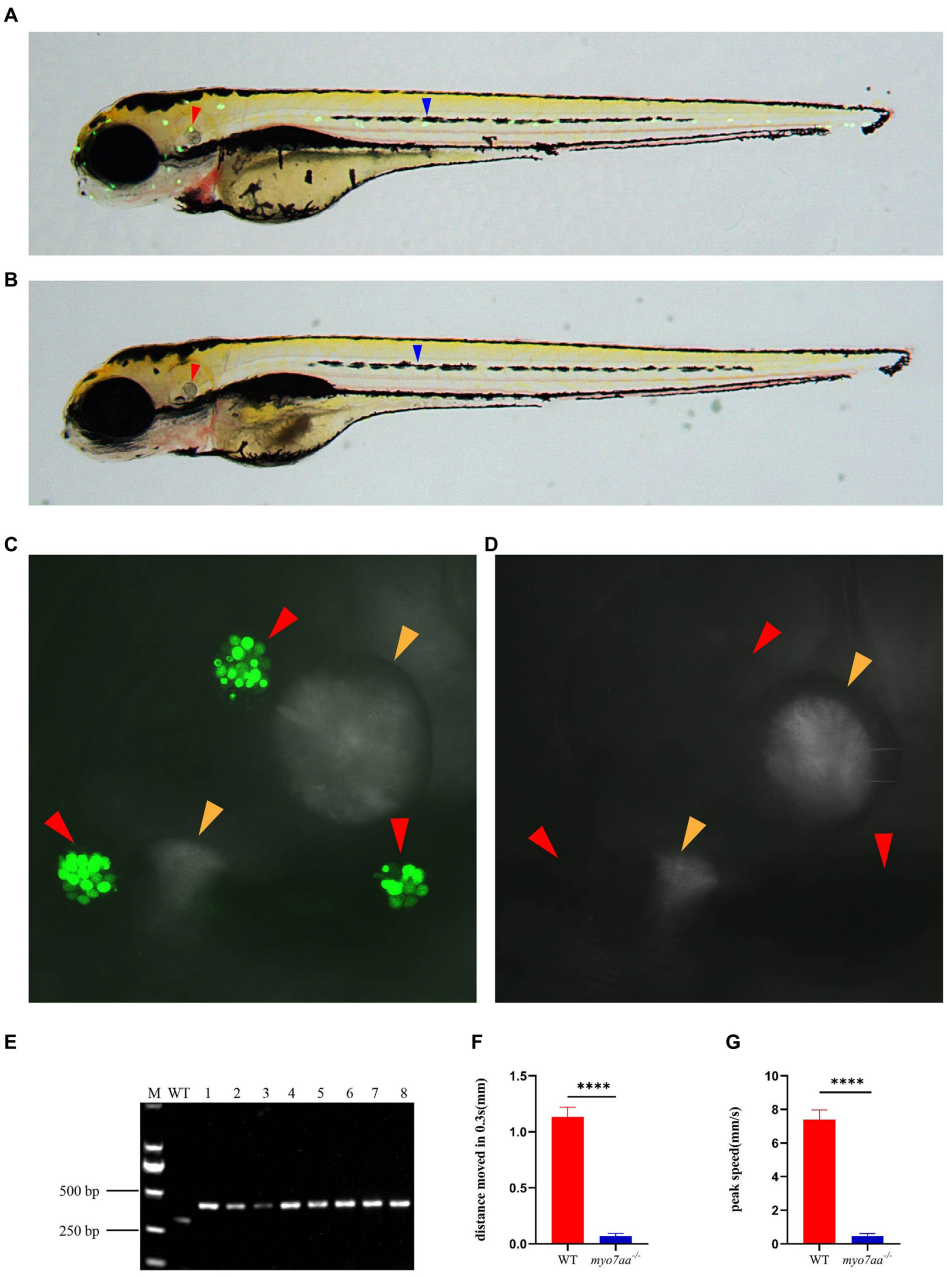
Loss of *myo7aa* causes hearing impairment. **(A,B)** YO-PRO-1 fluorescence staining results of wild-type and *myo7aa* mutant hair cells at 5 dpf, arrowheads indicate zebrafish inner ear (red) and lateral line hair cells (blue); **(C,D)** YO-PRO-1 fluorescent staining results of ear hair cells in wild-type and *myo7aa* mutant at 5 dpf, arrowheads indicate zebrafish inner ear hair cells (red) and otoliths (yellow). These results demonstrate the impact of *myo7aa* loss on the function of hair cells in the ear, leading to hearing impairment. Additionally, the results of genotype identification of embryos shown in **(B,D)** are presented. **(E)** Genotyping results of line2. M is DNA marker, WT indicates wild type, 1–8 are embryos shown in **(B,D)**. **(F,G)** The movement distance **(F)** and reaction speed **(G)** of wild type and *myo7aa* mutant within 0.3 s after acoustic stimulation at 5 dpf are measured; *n* = 20; mean with SEM; with *t*-test, **** indicates a *p*-value <0.0001.

Previous studies have shown that *myo7aa* is expressed in hair cells, which was confirmed by *in situ* hybridization. To investigate the role of *myo7aa* in hair cells, YO-PRO-1 staining was used to detect the abnormal function of hair cells. YO-PRO-1 is a cyanine dye that binds to DNA and fluoresces after entering the cell. Labeling of hair cells in the lateral line with YO-PRO-1 can be used as a reliable index to evaluate the viability of hair cells (Santos et al., 2006). At 5 dpf, the wild-type zebrafish larvae exhibited a bright and intact inner ear (Figure 3C) and lateral line hair cell signal (Figure 3A), whereas the *myo7aa* mutant embryos showed a complete lack of inner ear (Figure 3D) and lateral line hair cell fluorescence signal (Figure 3B). We confirmed the genotypes of YO-PRO-1 stained embryos, and the embryos without fluorescence signal were *myo7aa* homozygous mutant (Figure 3E). To confirm the effect of *myo7aa* on zebrafish hearing, we used YO-PRO-1 staining to screen *myo7aa* homozygous mutant larvae for startle response testing. Vibrators were connected beneath small dishes containing 20 wild-type or *myo7aa^−/−^ zebrafish* larvae, and the zebrafish larvae were stimulated with an acoustic wave at a frequency of 600 Hz and a pitch of 9 dB re.1 ms^−2^ for 30 ms. When frightened by sound waves, the average movement distance of wild-type zebrafish larvae was 1.134 mm per 0.3 s, while that of *myo7aa^−/−^* larvae was only moved 0.069 mm (Figure 3F; Table 1). Additionally, the reaction speed of *myo7aa^−/−^* larvae was much slower than that of the wild-type larvae. The maximum movement speed of the wild type zebrafish larvae was 7.388 mm/s, while the maximum movement speed of the *myo7aa^−/−^* larvae was only 0.468 mm/s (Figure 3G; Table 2). These results indicate that *myo7aa* gene knockout impairs the normal physiological function of hair cells and causes hearing impairment.

**Table 1:** The distance that WT and *myo7aa*^−/−^ moved in 0.3 s (mm).

WT	*myo7aa* ^−/−^
1.246629376	0.088650321
1.257808717	0.372098764
1.345192216	0.345624185
0.990287655	0
0.873995626	0
0.963666888	0.016598014
0.817987526	0
0.624802616	0
0.53748747	0
1.665803488	0.220574736
0.460196767	0.087435209
1.46030955	0
1.477797568	0
1.215035765	0.133896041
0.51661079	0
1.494572	0.01932545
1.308158848	0.039475638
1.744700811	0.043839207
1.405545456	0.012006092
1.269247475	0

**Table 2:** Peak speed of WT and *myo7aa*^−/−^ (mm/s).

WT	*myo7aa* ^−/−^
8.45734534	0.796974449
6.908661991	2.083611918
6.76307581	1.653264657
5.737211597	0
4.980088094	0
6.713954077	0
5.70996409	0
3.858035321	0
3.966787427	0
13.08830254	1.698379183
2.824149545	0.758577895
8.87464688	0
9.105157837	0
7.565859378	0.806514967
5.283165479	0
11.03621268	0.284118485
9.424169832	0.472286778
10.48937668	0.581109866
9.675427983	0.224615385
7.288911565	0

### The knockout of *myo7aa* resulted in the differential down-regulation of the Rho GTPase signaling pathway

**Figure 4:**
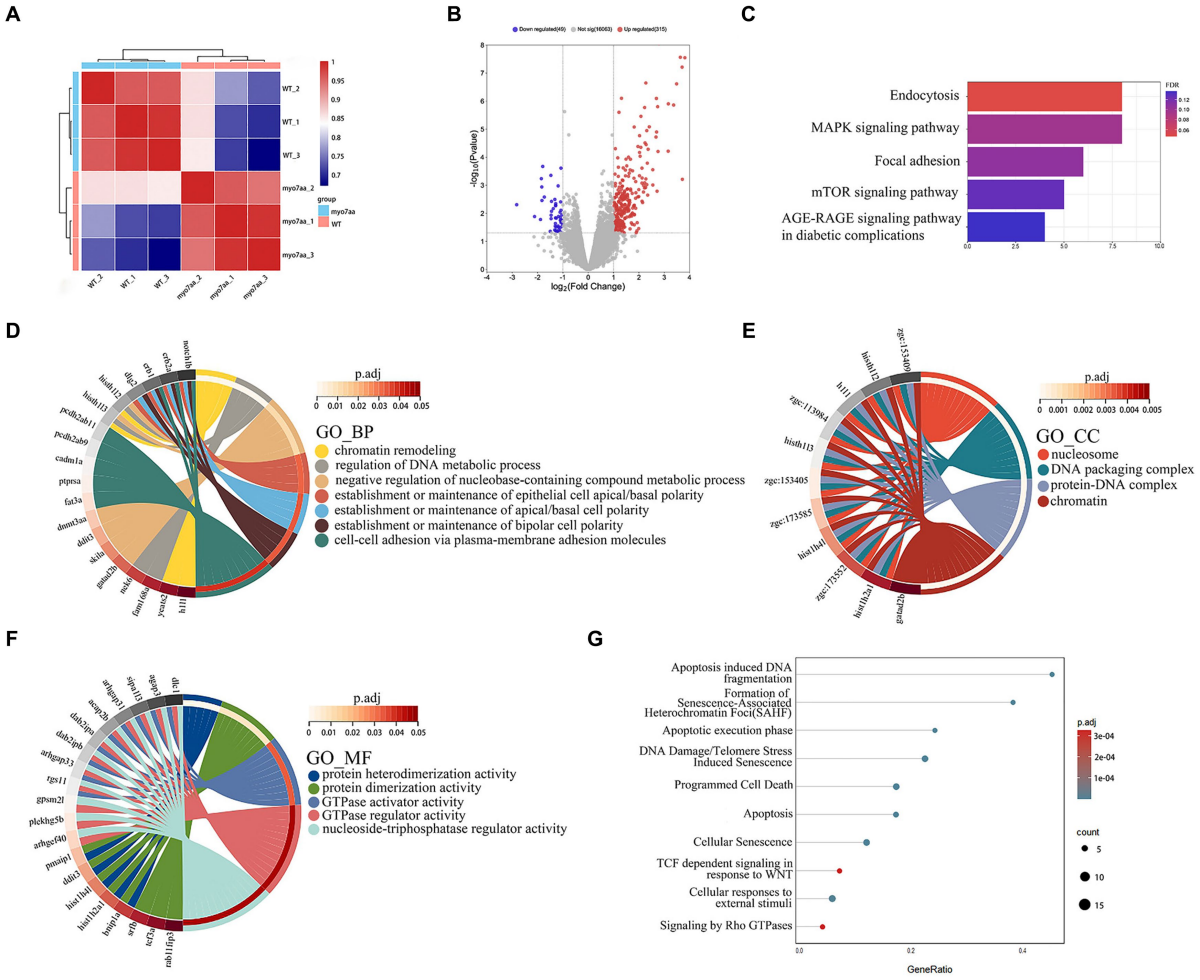
The results of RNA-seq analysis, **(A)** the Principal Component Analysis (PCA) diagram; **(B)** the volcano plot of differentially expressed genes. Red dots indicate upregulated genes. Blue dots indicate genes that are down-regulated; **(C)** the enrichment bar chart of gene function annotation analysis in the KEGG database. The bar length in the chart represents the number of genes. The bar color represents the p corrected value; **(D–F)** the GO database enrichment analysis results are provided for Biological process (BP), cellular component (CC), and molecular function (MF); **(G)** the Reactome database enrichment analysis results.

To further analyze the molecular mechanism of deafness caused by *myo7aa* deletion, we collected *myo7aa* knockout lines and TU embryos at 3 dpf for RNA-seq. Each group had three replicates, with 50 embryos per replicate. The principal component dimension reduction (PCA) analysis results showed that there was a clear separation between *myo7aa^−/−^* mutant and control. The samples in the two groups were clustered together, indicating that the samples within each groups were well grouped and correlated (Figure 4A). The differentially expressed genes (DEGs) results showed that the gene expression profile of *myo7aa*^−/−^ mutant was significantly changed. A total of 364 DEGs were screened between *myo7aa^−/−^* mutant and control, 49 of which were down-regulated and 315 were up-regulated (Figure 4B).

KEGG pathway annotation of DEGs was performed in the KEGG database, and the results of the enrichment analysis showed that all DEGs were significantly different in KEGG pathways (*p* < 0.05). The DEGs were mainly significantly enriched in the endocytosis and MAPK signaling pathway, and the genes involved in these pathways included *grk3*, *rab11fip3*, *agap3*, etc. (Figure 4C). The gene function annotation of DEGs in GO database showed that DEGs were involved in biological processes such as “regulation of DNA metabolism,” “establishment or maintenance of apical/basal cell polarity,” and “establishment or maintenance of bipolar cell polarity” (Figure 4D). Cellular components such as “nucleosome,” “DNA assembly complex,” “protein-DNA complex” and “nuclear chromatin” (Figure 4E). And molecular functions such as “GTPase-activating protein activity,” “GTPase-regulator activity” and “nucleic acid triphosphate regulator activity” (Figure 4F) were significantly enriched.

Gene function annotation of DEGs in Reactome database showed that the results were closely related to “DNA damage,” “telomere inhibition,” “apoptosis,” “programmed cell necrosis,” “Rho GTPase signaling” and so on (Figure 4G).

**Figure 5:**
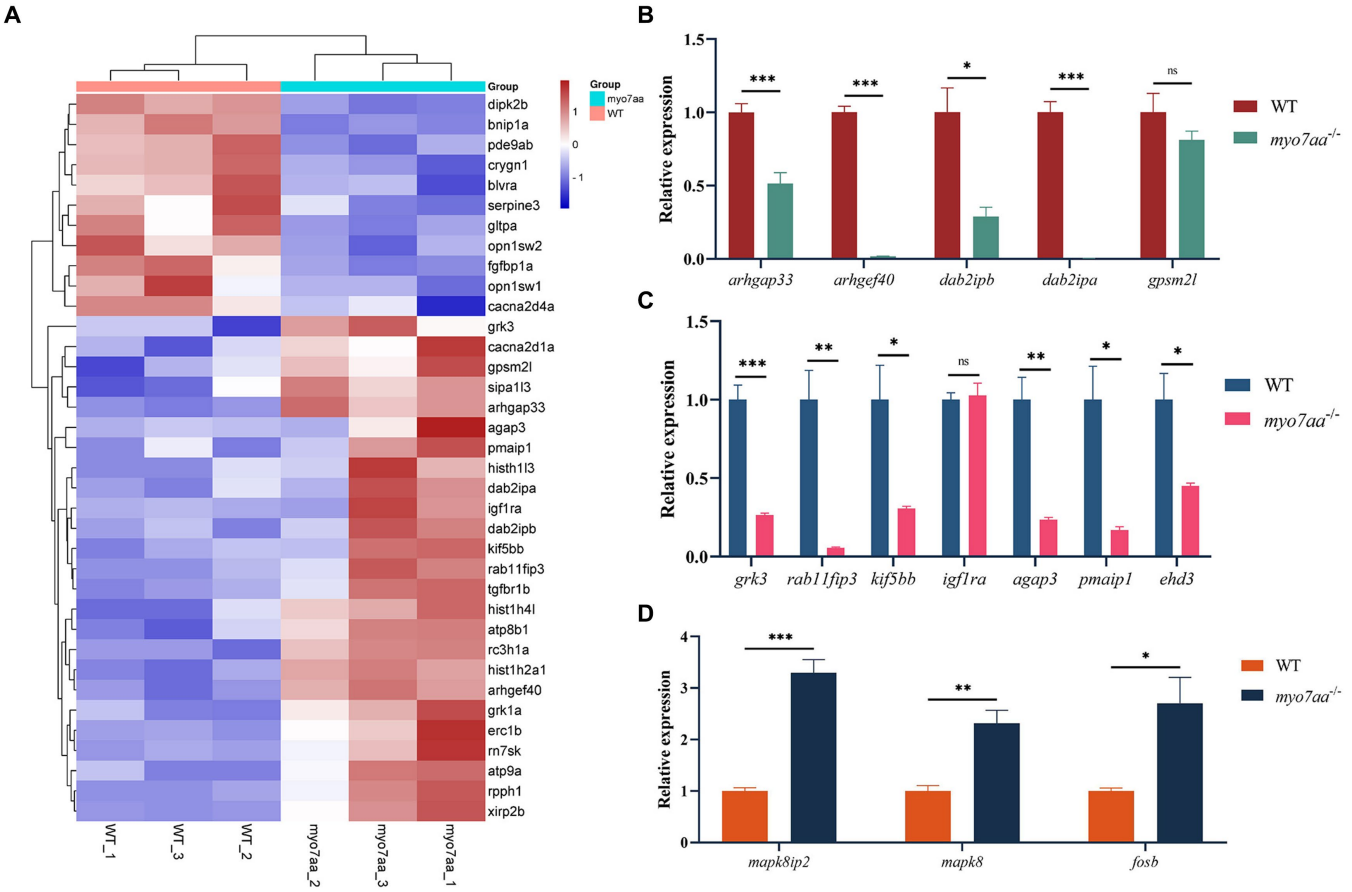
*myo7aa* regulates G-protein signaling. **(A)** The gene heatmap is depicted in **(A)**, while **(B–D)** display the relative expression of genes in the Rho GTPase signaling pathway **(B)**, cellular endocytosis pathway **(C)**, and MAPK signaling pathway **(D)** between WT and *myo7aa*^−/−^ at 3 dpf, ^*^p < 0.05, ^**^p < 0.01, ^***^p < 0.001 (*t*-test).

In order to further validate the results of transcriptome and bioinformatics analyses (Figure 5A), we collected control and mutant embryos at 3 days after embryonic development, 30 embryos were collected from each sample, with 3 replicates in each group. Total RNA was extracted and reverse transcribed using the PrimeScript™ RT reagent Kit (Takara), and then the qPCR primers for the relevant pathways were synthesized and subjected to qPCR experiments. qPCR validation results showed that Rho GTPase signaling pathway-related differential genes, such as *arhgap33*, a member of the sorted connexin family with the structural domain of Rho GTPase-activating protein (RhoGAP), and *dab2ipb*, a member of the Ras GTPase-activating protein family were significantly down-regulated. The guanine nucleotide exchange factor *arghef40*, which targets RhoA, and *dab2ipa*, a member of the Ras GTPase-activating protein family, are barely expressed in *myo7aa^−/−^* embryos (Figure 5B); endocytosis-associated genes, such as scaffolding protein family member *rab11fip3* and kinesin *kif5bb*, were significantly downregulated (Figure 5C); and MAPK signaling pathway-associated genes, such as *mapk8* (mitogen-activated protein kinase 2) and *mapk8ip2* (p38 family scaffolding protein) were significantly upregulated (Figure 5D).

These results suggest that a variety of cellular processes, including apoptosis and endocytosis, are affected in *myo7aa* deficiency. Notably, the disorder of Rho GTPase signaling system in *myo7aa^−/−^* zebrafish may lead to abnormal hair cell function and thus hearing damage.

### GTP compensation partially restored the defects in *myo7aa^−/−^* zebrafish

**Figure 6:**
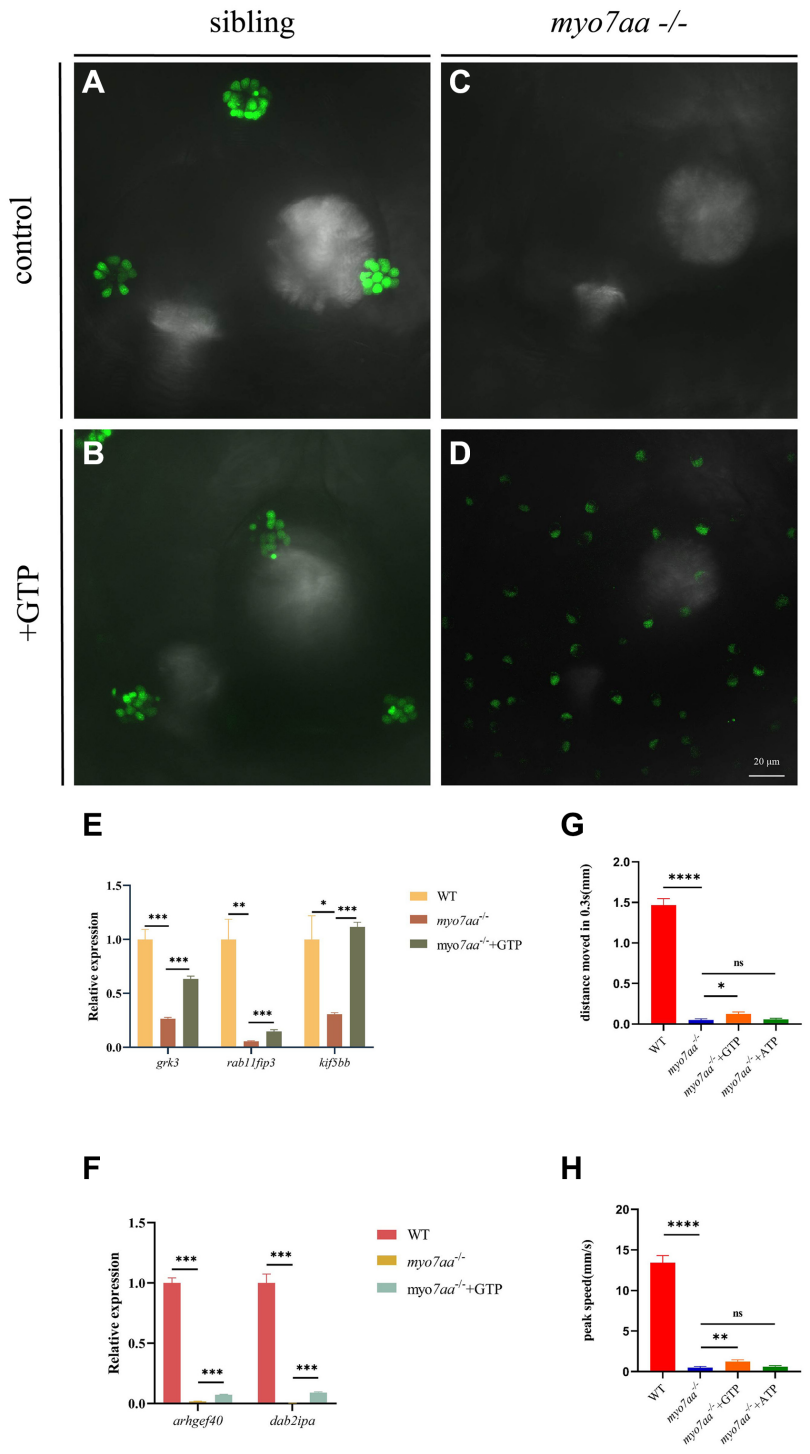
GTP compensation partially restored the hearing impairment caused by *myo7aa* deficiency. **(A–D)** YO-PRO-1 staining results of inner ear hair cells were observed in sibling **(A)**, *myo7aa*^−/−^ **(B)**, GTP-compensated sibling **(C)**, and *myo7aa*−/− **(D)** embryos at 5 dpf. **(E,F)** The relative expression of some genes in the cellular endocytic pathway **(E)** and G protein signaling pathway **(F)** is examined between WT, *myo7aa*^−/−^, and GTP-compensated *myo7aa*^−/−^ embryos at 3 dpf is examined. **(G,H)** The movement distance **(G)** and reaction speed **(H)** of wild WT, *myo7aa*^−/−^, and GTP-compensated *myo7aa*^−/−^ embryos are measured within 0.3 s after acoustic stimulation at 5 dpf are measured. *n* = 20; mean with SEM; with ANOVA, ****p < 0.0001, **p < 0.01, *p < 0.05, ^ns^*p* > 0.05.

Transcriptome and qPCR results showed that *myo7aa* deficiency resulted in dysregulation of GTPase activity, disordered GTP metabolism, and impaired cellular endocytosis. We next determined whether GTP compensation could rescue the loss of hair cell function in *myo7aa* homozygous mutants. After the YO-PRO-1 staining, we conducted GTP compensation experiments on sibling embryos, as previously described. YO-PRO-1 dye can enter inner ear hair cells and lateral line cells in sibling embryos, while the fluorescence signal is completely absent in *myo7aa^−/−^* embryos. Next, we added a 4 mM GTP solution to E3 water and incubated it for 6 h. The results showed that the fluorescent signal was still present in the hair cells of sibling embryos (Figures 6A,B). In contrast, the *myo7aa*^*−/*−^ group embryos showed partial restoration of the YO-PRO-1 signal in the inner ear and lateral line hair cells (Figures 6C,D). Subsequently, we collected the control and GTP-treated embryos for qPCR Experiment. The results showed that the expression of endocytosis related genes *grk3*, *rab11fip3*, and *kif5bb* was partially restored in *myo7aa^−/−^* embryos after GTP treatment (Figure 6E). The expression of *arghef40* and *dab2ipa* genes, encoding guanine nucleotide exchange factor and GTPase activating protein, was also restored (Figure 6F). The startle response experiment after GTP treatment showed that the group treated with GTP was more sensitive to sound stimulation compared to the *myo7aa* homozygous mutant. Additionally, there was a significantly improvement in both the movement distance and response speed to stimulation (ANOVA). When stimulated by sound waves, the average movement distance of wild-type zebrafish larvae was 1.466 mm per 0.3 s, whereas the *myo7aa^−/−^* larvae only moved 0.051 mm, and the GTP-treated larvae moved 0.124 mm (Figure 6G; Table 3). After sound stimulation, the maximum movement speed of wild-type zebrafish larvae was 13.427 mm/s, In contrast, the maximum movement speed of *myo7aa^−/−^* larvae was 0.488 mm/s, and the maximum movement speed of GTP-treated larvae was 1.241 mm/s (Figure 6H; Table 4). These results suggest that knockout of *myo7aa* leads to dysfunction of GTPase signaling, which is caused by disrupted GTPase activity. Additionally, the application of GTP can partially restore hair cell function.

**Table 3:** The distance that WT, myo7aa^−/−^, myo7aa^−/−^ + GTP and *myo7aa*^−/−^ + ATP moved in 0.3 s (mm).

WT	*myo7aa* ^−/−^	*myo7aa*^−/−^ + GTP	*myo7aa*^−/−^ + ATP
1.479563771	0.151878088	0.216222951	0
0.929838655	0.086579736	0.269223803	0.183025995
1.159282707	0.09312932	0.427388607	0.090527133
1.880445683	0.25755212	0.110293449	0.056723733
1.497505607	0	0.150355746	0.161637362
0.666928134	0	0.105524258	0
1.404930781	0	0	0
1.451113935	0.121752522	0.087257573	0.078741942
0.995271604	0	0.217632238	0.0595489
1.136841589	0.083762853	0.04298246	0.078159429
1.488803471	0	0.065924649	0
1.914394534	0	0	0
2.019293869	0	0.135768709	0.056173132
1.726803624	0	0.074124044	0.067964906
1.9041604	0.094392598	0.07621195	0
1.519701169	0	0.079651442	0
1.709662817	0	0	0.18335076
1.51480562	0.063707934	0.054171181	0.121419007
1.615613072	0	0.103525756	0
1.306896249	0.065271785	0.273409573	0

**Table 4:** The peak speed of WT, myo7aa^−/−^, myo7aa^−/−^ + GTP and *myo7aa*^−/−^ + ATP (mm/s).

WT	*myo7aa* ^−/−^	*myo7aa*^−/−^ + GTP	*myo7aa*^−/−^ + ATP
9.154635129	1.827087262	2.328992724	0
5.967400416	0.616997863	2.523930397	1.454499906
14.09166537	0.86163565	3.439314147	0.781170157
20.33487777	1.859725036	0.943721652	0.639703038
16.24149396	0	1.642576539	1.380763782
7.507319751	0	1.535549913	0
14.34551186	0	0	0
13.28417714	1.556017636	0.861930916	1.710114294
11.6280096	0	3.002235641	0.498602107
7.810630258	0.801196285	0.545949365	1.222500831
15.44708469	0	0.635082683	0
18.66212143	0	0	0
17.80184275	0	1.403907104	0.896027072
18.72085993	0	0.874014766	0.4945786
13.95008096	1.112100428	0.683708766	0
15.14133996	0	0.377279527	0
10.35414941	0	0	1.726815385
12.84292868	0.504555846	0.795349596	1.009613959
13.65233449	0	1.242686841	0
11.59888494	0.619038197	1.985908507	0

### ATP compensation partially restored the defects in *myo7aa^−/−^* zebrafish

**Figure 7:**
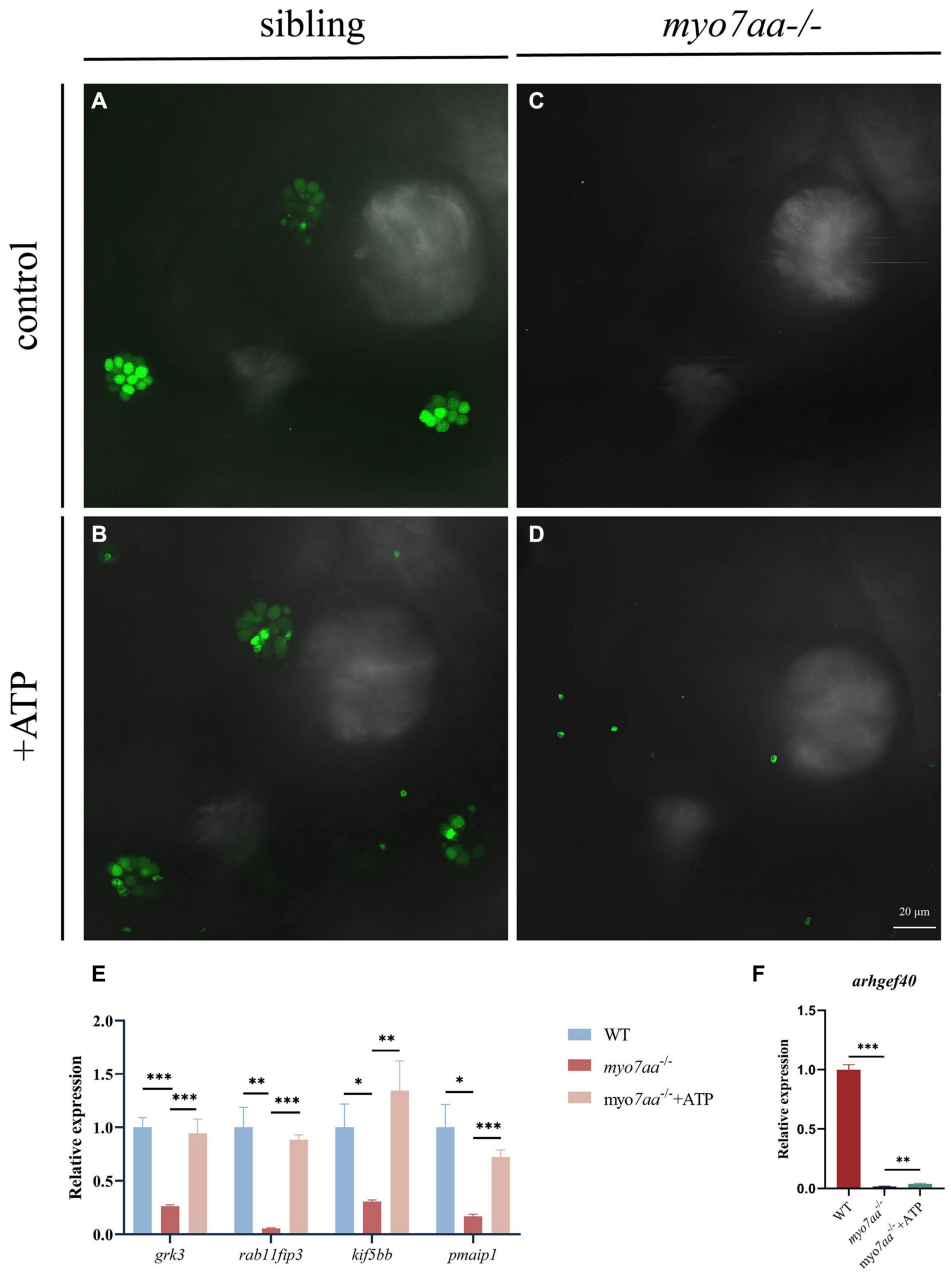
ATP compensation partially restored the hearing defects caused by *myo7aa* deficiency. **(A–D)** The YO-PRO-1 staining results of inner ear hair cells were observed in sibling **(A)**, *myo7aa*^−/−^ **(B)** and ATP-compensated sibling **(C)**, *myo7aa*^−/−^ **(D)** embryos at 5 dpf. **(E,F)** The relative expression of some genes in the cellular endocytic pathway **(E)** and G protein signaling pathway **(F)** was compared between WT, *myo7aa*^−/−^ and ATP-compensated *myo7aa*^−/−^ embryos at 3 dpf.

**Figure 8:**
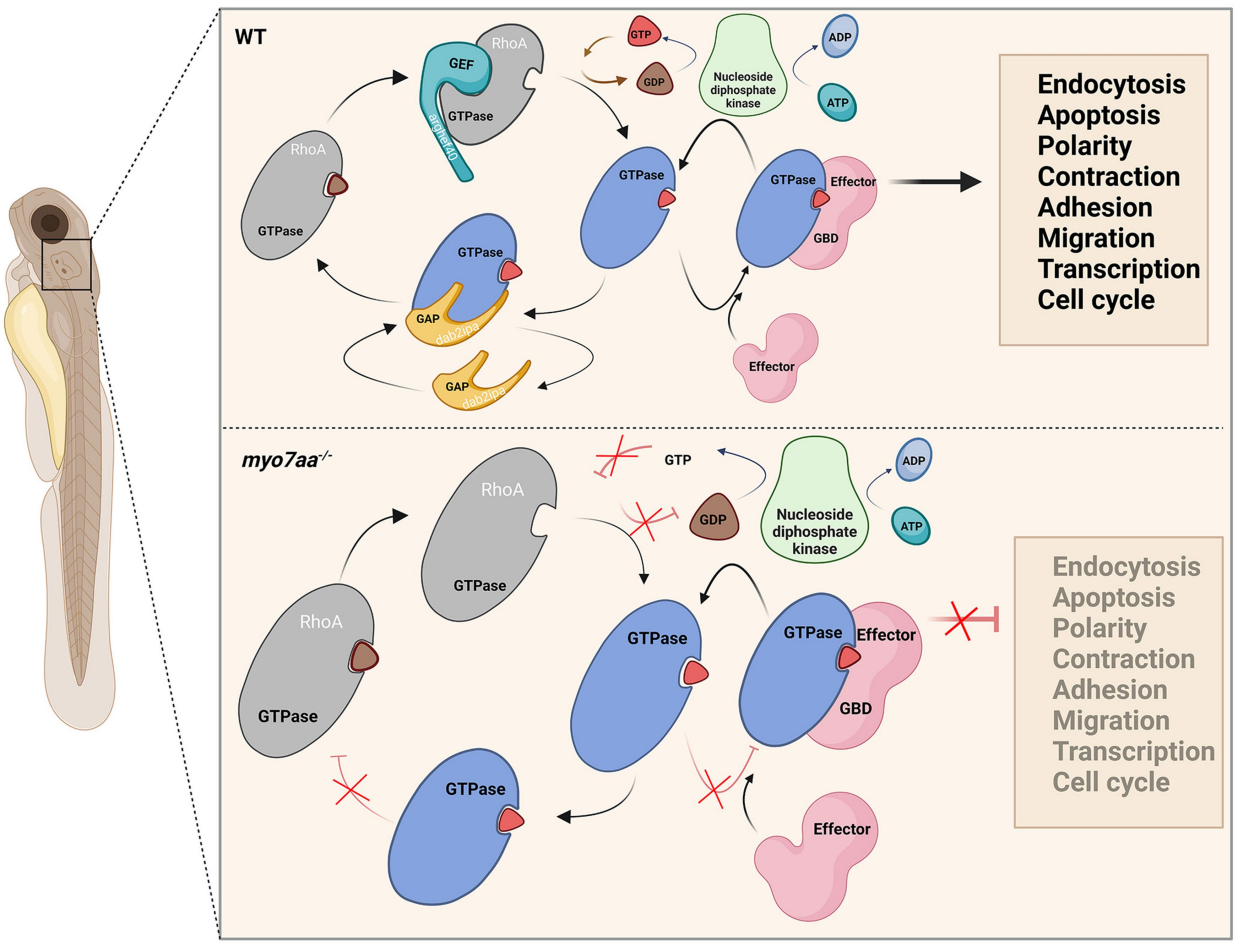
Loss of *myo7aa* leads to impaired Rho GTPase signaling, resulting in hearing impairment (image created at BioRender.com).

Nucleoside diphosphate kinase (NDK) can transphosphorylate GDP and ATP near the cell membrane and G protein to generate GTP and ADP (Piacentini and Niroomand, 1996). To verify whether ATP affects the G-protein signaling process, ATP compensation experiments were performed after the YO-PRO-1 staining experiment, as described previously. Zebrafish embryos at 5 dpf were treated with 3 mM ATP in combination with YO-PRO-1 and observed under a fluorescence microscope after 6 h. Fluorescence signals were still observed in the inner ear and lateral line hair cells of Sibling embryos (Figures 7A,B), and YO-PRO-1 fluorescence signals were partially recovered in *myo7aa*^*−/*−^ embryos (Figures 7C,D). The expression of endocytosis related genes *grk3*, *rab11fip3*, *kif5bb* and *pmaip1* was partially restored (Figure 7E), and the mRNA expression level of arghef40 was also restored (Figure 7F). These results indicate that the application of ATP could partially restore the function of hair cells in *myo7aa* ^−/−^embryos. The startle response experiment conducted after ATP treatment revealed that neither the ATP treatment group nor the *myo7aa* homozygous mutant showed sensitivity to acoustic stimulation. When subjected to by acoustic stimulation, *myo7aa^−/−^* larvae moved a distance of 0.051 mm, while the ATP treatment group moved a distance of 0.057 mm (Figure 6G; Table 3). When stimulated by sound waves, the maximum movement speed of *myo7aa^−/−^* and ATP-treated larvae was 0.488 mm/s and 0.591 mm/s, respectively (Figure 6H; Table 4). There was no significant difference in movement distance and reaction speed between the two groups (ANOVA) (see Figure 8).

## Discussion

Previous studies have shown that MYO7A is the causative gene for Usher 1B syndrome, recessive nonsyndromic deafness (DFNB2), and dominant nonsyndromic deafness (DFNA11). In *myo7aa^−/−^* zebrafish, hair cell function is impaired, but the downstream regulatory network is not well understood. In this study, we generated a *myo7aa* knockout zebrafish line to investigate the molecular functions of *myo7aa* in hair cells. To compare the transcriptome data of wild-type and *myo7aa* knockout zebrafish, We analyzed the differentially expressed genes. We found that these genes were mainly involved in pathways such as “nucleosome assembly,” “protein-DNA complex,” “establishment or maintenance of epithelial cell apex,” “establishment or maintenance of bipolar cell polarity,” “establishment or maintenance of apical/basal cell polarity,” “GTPase-activating protein activity,” “GTPase regulator activity,” “nucleoside triphosphatase regulator activity,” “endocytosis exocytosis,” “autophagy” and other pathways were enriched. The genes involved in these pathways, such as *grk3*, *rab11fip3*, and *agap3,* play crucial roles in the regulation of various biological processes.

Endocytic transport plays a crucial role in the establishment and maintenance of cell polarity. Polarized cells, especially highly polarized cells such as hair cells, rely heavily on the proper organization of their intracellular components to achieve their function (Nakazawa et al., 2016). Subcellular localization of organelles, proteins, and RNA is essential for the compartmental function of cells. Rab11 GTPase has been shown to be a master regulator of endosomal trafficking via recycling, which is required to establish and maintain epithelial polarity (Jing and Prekeris, 2009). *Rab11-FIP3* is a member of the Rab11-FIP family. Rab11-FIP is a family of scaffold proteins that play a major role in mediating endocytic recycling in a variety of cells (Horgan et al., 2004, 2007; Inoue et al., 2008). Kinesin superfamily proteins are a class of kinesin proteins. Kif5s has a conserved carved structure consisting of an N-terminal globular head/motor domain and a C-terminal globular tail domain connected to the coiled-rich stem region via a neck domain. The motor domain is responsible for ATP hydrolysis and microtubule binding, allowing the complex to move along microtubules (Campbell and Marlow, 2013). Kif5s has been implicated in a number of transport processes including retrograde transport of vesicles from the Golgi to the endoplasmic reticulum (ER) (Lippincott-Schwartz et al., 1995), anterograde transport of lysosomes to the plasma membrane (Nakata and Hirokawa, 1995), pigment dispersion in melanocytes (Hara et al., 2000), and anterograde axonal transport of organelles, proteins, vesicles, and RNA in neurons (Hirokawa et al., 2010). Eps15 homeodomain-containing protein 3 (EHD3) is an endocytic trafficking regulator (Juszczak and Stankiewicz, 2018) that controls trafficking to and from the endocytic recycling compartment (ERC) to the plasma membrane (Naslavsky et al., 2009; Cabasso et al., 2015) and is required for the stabilization of tubular recycling endosomes (Bahl et al., 2016). ARHGAP33 (also known as SNX26, TCGAP or NOMA-GAP) is a member of the sorting junction protein (SNX) family. Mediating interactions with phosphatidylinositol via the conserved PX domain, SNX proteins normally regulate membrane protein sorting in the Golgi apparatus and endosomes, where they are localized (Liu et al., 2006; Kim et al., 2013; Nakazawa et al., 2016).

*igf1r* is essential for the early development of zebrafish. *igf1ra encodes* the insulin-like growth factor 1 receptor, which is expressed in the ear capsule, an inner ear (auditory) and vestibular (balance) organ of zebrafish (Li et al., 2014).

Purine nucleotide GTP is involved in a variety of cellular processes, including RNA and DNA synthesis, G-protein signaling, protein biosynthesis, gluconeogenesis, and tubulin formation (Wolff et al., 2022). GTPase activity is essential for accelerating synaptic vesicle recruitment (Watanabe et al., 1999). Rho GTPase regulates actin dynamics near the cell membrane, and GTP plays an important role in cellular endocytosis (Yamashita et al., 2005; Xu et al., 2008). AGAP3 is a member of a family of proteins containing functional ArfGAP domains and GTpase-like domains (AGAP1-3) with bifunctional enzyme activity (Oku and Huganir, 2013). Solo*(arghef40)* is a guanine nucleotide exchange factor that targets RhoA and regulates cell morphology by interacting with keratin filaments (Nishimura et al., 2018). DOC-2 / DAB-2 interacting protein (Dab2IP) is a member of the Ras gtpase activating protein (GAP) family (Lee et al., 2012; Qiao et al., 2013). Guanine nucleotide exchange factors (GEFs) can facilitate the exchange of GDP-GTP bound by small guanine nucleotide-binding proteins (G proteins), while GTPase activating proteins (GAPs) facilitate the hydrolysis of GTP by G proteins to GDP (Fujiwara et al., 2018). Together, they regulate the activity of G proteins. Upon binding to GTP, the G protein is activated and can bind to other proteins and trigger downstream signaling targets. Upon binding to GDP, the G protein loses its activity and is unable to bind to its target proteins, resulting in impaired RNA and DNA synthesis, G protein signaling, protein biosynthesis (Jewett et al., 2009), gluconeogenesis, and tubulin formation (Muraoka and Sakai, 1999; Pickett and Raible, 2019). GRK3 (G protein-coupled receptor kinase 3) is highly expressed in sensory epithelial cells and mediates the agonist-dependent phosphorylation and uncoupling of many G protein-coupled receptors (Peppel et al., 1997).

Both GTP and ATP compensation could partially rescue the defects in *myo7aa* knockout zebrafish. Therefore, we hypothesized that knockout of *myo7aa* results in decreased expression of GTPase activator protein *dab2ip* and guanine nucleotide exchange factor *arghef40*, which prevents G protein from properly binding and activating GTP and leads to Rho GTPase signaling dysfunction. It affects RNA and DNA synthesis, protein biosynthesis, endocytosis and exocytosis, and the establishment or maintenance of cell polarity. Compensating GTP can increase the concentration of GTP in hair cells, accelerate the exchange of GTP and GDP, and lead to the activation of Rho GTPase, so as to transmit signals normally. When ATP is compensated, nucleotide diphosphate kinase catalyzes the regeneration of GTP in GDP due to the presence of ATP, and at the same time converts ATP to ADP (Aamodt and Williams, 1984; Dewulf et al., 2022), making Rho GTPase bound to GTP into an activated state, and G protein signaling can be partially restored (Figure 8). Since ATP affects G-protein signaling by promoting GTP production, ATP compensation did not significantly restore hearing in *myo7aa* homozygous mutant embryos.

## Data availability statement

The datasets presented in this study can be found in online repositories. The names of the repository/repositories and accession number(s) can be found at: https://dataview.ncbi.nlm.nih.gov/object/PRJNA1086823?reviewer=atbodt8tbhj1tqo6bpv6l7n3cq.

## Ethics statement

The animal study was approved by Biomedical Research Ethics Committee of Hunan Normal University. The study was conducted in accordance with the local legislation and institutional requirements.

## Author contributions

BX: Data curation, Investigation, Project administration, Validation, Visualization, Writing – original draft. JL: Data curation, Formal analysis, Software, Visualization, Writing – original draft. JJ: Investigation, Validation, Writing – original draft. TZ: Writing – original draft. LL: Data curation. DX: Funding acquisition, Resources, Writing – review & editing. GZ: Writing – original draft, Resources. LX: Writing – review & editing. KZ: Resources, Writing – original draft. DL: Resources, Writing – review & editing. JG: Resources, Writing – review & editing. XC: Writing – review & editing. RL: Funding acquisition, Investigation, Resources, Writing – review & editing. HX: Conceptualization, Formal analysis, Funding acquisition, Methodology, Resources, Writing – review & editing.

## Footnotes

^1^ http://smart.embl.de/
