# Supplementary material for: Zebrafish myo7aa affects congenital hearing by regulating Rho-GTPase signaling
Source: Front Mol Neurosci. 2024 Jul 15;17:1405109. doi: 10.3389/fnmol.2024.1405109 (PMC11287254; doi:10.3389/fnmol.2024.1405109)
Supplement: Supplementary file 1 [file Table1.DOCX]

| Number | Gene name | Forward primer | Reverse primer |
| --- | --- | --- | --- |
| 1 | *mapk8ip2* | CGCCCGACCACTCTAAACTT | GACTTCCGTGGAGACACGTT |
| 2 | *mapk8* | CCACCAGCGATCACGGATAA | TTGTTCGCTCCTCCCATTCC |
| 3 | *fosb* | CGGCAGCTACATCCCCTCAT | GCAAGAAGCGAGGGTGAGTT |
| 4 | *arhgap33* | GAGCAGCTGGCAATAAAGGC | GTCTTACCCTGGCAGGACAC |
| 5 | *arhgef40* | ATACAGGCTTCACACGCCAA | AGCAGGGGCAGGTAGTTTTC |
| 6 | *dab2ipa* | CCAGGGATGGATGAGGGTCA | TTCTGGTTTGTGTGCTCTGGT |
| 7 | *dab2ipb* | GCACACACAAGCTGAAAAGGC | CAGTCGGGCCTGATACTCCA |
| 8 | *gpsm2l* | ATCGGCATGACCTCACACTC | TGGCAACAAACTGCTGCTTC |
| 9 | *pmaip1* | CGAAGAAAGAGCAAACCGCT | CATCGCTTCCCCTCCATTTGT |
| 10 | *grk3* | GGAGAGTCTCGGCAAAACCT | TAGTTCTGCCCTTGCCGATG |
| 11 | *rab11fip3* | ATTTCCTTCAGTCGGAGGCG | GCAGCAACACCTTGTCTGTG |
| 12 | *kif5bb* | TGCCCGGCCTCAAATTTACT | CCTTAGACGCCTCGTTCTCC |
| 13 | *igf1ra* | CTGAAGACCGATCCTACCGC | GAAGGGCCGAAGGTTGTGTA |
| 14 | *agap3* | AATTACCAGTCGAGCGTCCC | CTGATGGCTGCTGAGTTGGA |
| 15 | *pmaip1* | CGAAGAAAGAGCAAACCGCT | CATCGCTTCCCCTCCATTTGT |
| 16 | *ehd3* | GGTTGGGTACCGATGACAGG | GTCGTGGGTTCTGGACCAAT |

Table S1 Primer sequences for qPCR
